# The link between impact-induced tensile strain and dendritic spine morphology in porcine brain tissue

**DOI:** 10.1371/journal.pone.0318932

**Published:** 2025-02-24

**Authors:** Brendan Hoffe, Gia Kang, Hannah Thomson, Rohan Banton, Thuvan Piehler, Oren E. Petel, Matthew R. Holahan

**Affiliations:** 1 Departement of Neuroscience, Carleton University, Ottawa, Ontario, Canada; 2 Department of Mechanical and Aerospace Engineering, Carleton University, Ottawa, Ontario, Canada; 3 U.S. Army Research Laboratory, Aberdeen Proving Ground, Maryland, United States; PLOS: Public Library of Science, UNITED KINGDOM OF GREAT BRITAIN AND NORTHERN IRELAND

## Abstract

Brain tissue as a material presents unique properties with a multitude of cell types and densities, varying degrees of axonal fiber diameters and blood vessels. These neural components are contained within a very viscous environment that upon impact, can result in a variety of tensile, compressive and rotational forces. The depths of the sulcus appear to be particularly vulnerable to biomechanical forces following an impact. The movement and subsequent forces loaded on to the brain have been shown to produce a variety of biomechanical responses that impair neurophysiological functioning at the cellular level. We recently reported a decrease in microtubule associated protein 2 (MAP2) within the depths of the porcine sulcus in an *ex vivo* model, along with elevated tensile strain in this region within 1 hour after impact. In the current work, using the same impact model, we explored whether changes in spine morphology and density occurred within the same timeframe following impact. The Golgi-Cox method was used to visualize dendritic spine morphology. Cortical pyramidal neurons within the depths and the arms of the sulcus were reconstructed. One hour after impact, there was a change in the distribution of spine type resulting in an increased proportion of mushroom-type spines compared to nonimpacted tissue. The increased proportion of mushroom-type spines was proportional to tensile strain measurements in the apical dendrites. These results demonstrate the sensitivity of dendritic spine morphology to tensile strain within the porcine cortex and suggest a state of hyperexcitability during the hyperacute phase following an impact.

## Introduction

Traumatic brain injury (TBI) typically results from the application of an impulsive load to the head or body, leading to brain tissue deformation and subsequent neurological dysfunction [1]. TBI is a multifactorial consequence, with a wide range of mediators determining the severity and overall outcome associated with the impact. Within the gyrified brain, little is known about the relationship between impulsive load and cellular function immediately following impact [2] due to the dearth of animal models that share a similar brain organization as humans (i.e., gyrified cortex) [3]. While cellular-level changes due to abrupt increase in tissue strain beyond an acceptable threshold is thought to be the primary mechanism of injury [4–6], the strain threshold for tissue-level damage, as well as a description of the pathophysiology of this damage, remain elusive.

Analysis of post-mortem human brain samples has revealed that long-term pathological consequences of repeated impacts to the brain manifest in the deep sulcal regions of the cortex [7–9]. The development of appropriate models to investigate the changes in the gyrified brain is crucial for understanding why there is a particular vulnerability within the depths of the sulcus after an impact. To more accurately reflect the gyrified human brain, we have employed an *ex vivo* model using pig brains. With this model, we reported a decrease in microtubule associated protein 2 (MAP-2) within the depths of the porcine sulcus 1 hour after experimental drop impact [10]. Considering how MAP-2 dissociates from the microtubule bundles following excitotoxic conditions, the aim of the current study was to investigate whether changes in synaptic structure, represented by spine morphology, might also take place after impact.

During the hyperacute phase (minutes to hours after an impact), affected neurons undergo impaired signaling and hyperexcitable states due to ionic imbalance [11–13], impaired inhibitory signaling [14,15], and the onset of excitotoxic conditions [16–18]. This initial cellular dysfunction after impact can worsen if left unresolved, with prolonged maladaptive responses associated with the onset of neurological dysfunction [19], potentially setting off a cascade associated with the development of neurodegenerative diseases [20].

Along the dendrite, spines represent the anatomical locus of communication between two neurons. Spines are known to periodically change their morphology based on activity-related inputs. Recent reports [21–23] used controlled cortical impact, as well as blast-induced impacts within rodents to investigate synaptic changes within the first 24-72 hours post-impact in various brain regions. Results showed that impact was associated with an increase in the number of mushroom-like shaped dendritic spines suggestive of an elevation in excitatory connections. The synaptic changes observed were due to the upregulation of remodeling proteins within the extracellular matrix associated with hyperexcited and excitotoxic conditions [23]. The changes to dendritic spines within the gyrified cortex during the hyperacute phase after impact have yet to be examined.

The synapse is regarded as the focal point for the continuation of the hyperexcitable state during the hyperacute phase. Post-impact, hyperexcitability results from a multitude of cellular factors on both the pre- and post-synaptic side. Given that synapses are the primary sites of communication between neurons, understanding the synaptic changes that occur shortly after impact could help elucidate activated pathways that promote neuropathology associated with brain injury.

In the current work, we investigated morphological dendritic spine changes within the hyperacute phase that were associated with strain placed on to the gyrified porcine brain. A change in the proportion or density of mushroom-type spines would lend support to an overall excitatory environment during the hyperacute phase after impact. Our model also allowed us to directly compare tensile loads in different areas of the folded cortex to better understand the relationship between strain values and biological outcomes associated with brain impacts.

## Methods

### Porcine brain removal

The method for porcine brain removal from the skull was as previously described [10]. A total of 3, 10–12-month Yorkshire pig heads were collected (1 head per month) immediately post-mortem from a local abattoir, placed on ice, and transported back to the laboratory (approximately 1 hour travel time). Brains were removed in approximately 15 minutes and placed into ice cold artificial cerebral spinal fluid (aCSF) for 1 hour. Coronal slabs (5-mm-thick) were cut starting at 3.0 cm from the front of the brain and placed into a compartment filled with fresh aCSF. Two slabs from each brain were designated as experimental (impact) and two from the same brain were designated as control. This resulted in 6 impacted slabs and 6 control slabs. Two control and two impacted slabs were always collected from the same brain to control for any variation in the method of tissue collection (e.g., euthanasia, travel time, brain extraction). The starting distance for slab collection was chosen to be consistent between brains and allow sufficient brain tissue to implant markers to avoid the risk of overlapping marker placement. No animal ethics were needed for the current study. Biosafety and Biohazards was approved by Carleton University Biohazards Committee (Biohazards Certification 109314).

### Marker insertion

Radio-opaque cylindrical markers were inserted into coronal brain slabs to track tissue deformation during impact. Markers with two different sizes, 1.5 mm ×  1.5 mm and 1.0 mm ×  1.5 mm (diameter x height), were inserted at a depth of 1.5 mm into the white and grey matter of the brain slabs. The markers were 60-40 weight % mixture of barium sulphate and a thermoplastic gel (Gelatin #4, Humimic Medical). These markers were optimized for brain tissue characterization through a prior parametric marker sensitivity analysis [24] and have been previously used to track cadaveric brain motions [25]. Their density and stiffness have been tuned to minimize interference with brain tissue deformation. Marker placement was limited to the top layer of the tissue to leave the remaining tissue intact for histological processing. After inserting markers into the slabs, both those subjected to impact and the control (sham) specimens, tissue was returned to the bubbling aCSF.

### Drop impact

The drop impact test procedures used in this investigation followed the methods used in prior work [10]. Following marker insertion, two brain slab specimens were inserted within elastomer encasements (Sylgard 527:184 in a 5:1 ratio [26]) and sandwiched between two acrylic plates for drop impact. Each encasement measured 100 mm x 80 mm x 22 mm once sealed. The sealed encasements were dropped from a height of 0.9m onto a steel anvil, with an impact velocity of approximately 3.8 m/s. All slabs were impacted in the vertical plane. This more accurately reflects what happens *in vivo*. *In vivo*, there is rarely the same distance from impact to all brain regions. Often brain regions distant from the point of impact show the greatest pathology (consider the medial temporal lobe that shows pathology in cases of chronic traumatic encephalopathy where the impact is often far from that neural region). The deformation of the *ex vivo* brain tissue resulting from the impact was imaged via a high-speed X-ray cinematography system (HSXR) at 7500 fps. X-ray imaging was selected due to the elastomeric confinement of the brain tissue slab, which could distort optical surface measurements. The utility of the encasement design, drop impact condition, and strain measurement approaches were confirmed in an earlier investigation involving a porcine brain tissue surrogate [27]. The drop condition is not meant to replicate any specific injurious event, but rather a simple technique to induce a strain response in the tissue that can be easily measured and used to observe tissue-level changes. After the impact event, both control and impacted slabs were placed into bubbling aCSF for 1 hour then fixed for tissue processing.

### Particle tracking and strain analysis

Mosaic Particle Tracker [28], an add on to Fiji [29] distributed by ImageJ [30], was used to track the motion of individual markers via a point tracking algorithm. Prior to marker tracking, image distortion of the captured radiographic images was corrected using an in-house algorithm. The images were post-processed to increase marker contrast, as required by the tracking algorithm. The motion patterns of the individual markers were tracked using Mosaic and used to calculate the maximum principal strain (MPS), minimum principal strain (mPS) and maximum shear strain. A kernel radius of 3, a cut-off of 0, an intensity percentile of 2, a link range of 2, a displacement of 6, and Brownian dynamics were used.

**Fig 1:**
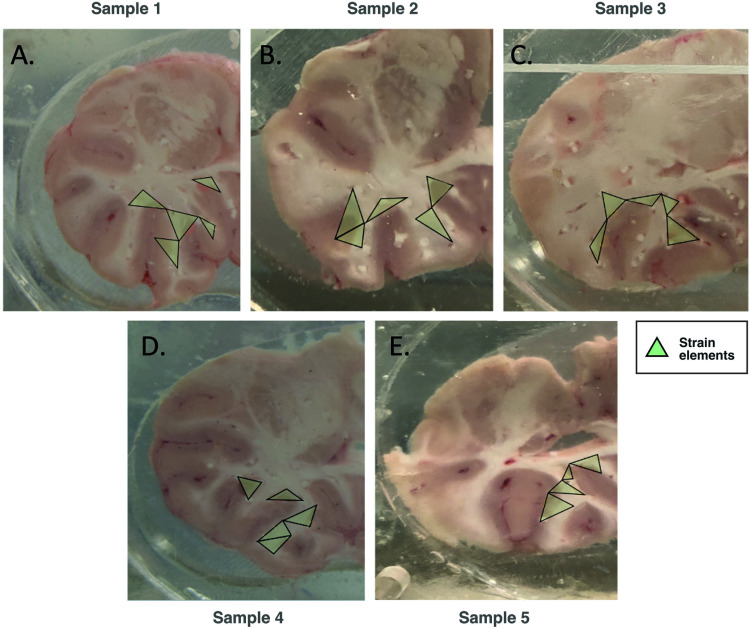
Impacted porcine brain slabs used for strain data collection within elastomer encasements, focused on areas containing the relevant ROI and 3-node triangular elements. On average, a slab would contain five ROIs for neuron reconstruction analysis and a corresponding triangular strain element per ROI. Indicated are the strain elements (in green) in various locations within the arm and apex of the porcine sulcus.


The displacements of the markers installed within the tissue specimens were tracked in the HSXR images and used to calculate the local strain response. The 2D Green-Lagrangian strains were calculated from 3-node triangular elements chosen such that they encompass the sulcal arm and apex found within the tissue region near of the point of drop impact. The elements were chosen such that the distances between their centroids and the points of impact were relatively consistent. The displacements of the markers/nodes of the elements were used to define the deformation gradient tensor (***F***) based on the displacement vectors, *u* and *v* for a set of 3-node triangular elements centered in the regions of interest (i.e., sulcus and gyrus). As the markers were inserted prior to impact, the choice of triangular elements was constrained by marker placement. The 3-node triangular strain elements are shown in Fig 1.

$$\left[\begin{array}{c} u\left(x,y\right)\\ v\left(x,y\right) \end{array}\right]=\left[\begin{array}{ccc} a_{o} & a_{1} & a_{2}\\ b_{0} & b_{1} & b_{2} \end{array}\right]\left[\begin{array}{c} 1\\ x\\ y \end{array}\right].$$ (1)

The right Cauchy-Green deformation tensor (C) can then be defined from the deformation gradient tensor as:


$$C=F^{T}F.$$ (2)

The Green-Lagrangian strain tensor (***E***) is defined as:


$$E=\frac{1}{2}\left(C-I\right).$$ (3)

The MPS (ε_1_) and mPS (ε_2_) were defined as the maximum and minimum Eigen values of the strain tensor, respectively. The maximum shear strain within each element was calculated as:


$$\varepsilon_{MSS}=\frac{1}{2}\left(\varepsilon_{1}-\varepsilon_{2}\right).$$ (4)

The strain responses were filtered using a 2nd-order Butterworth filter at 1 kHz to remove high-frequency noise due to the marker tracking algorithms.

### Golgi-Cox staining

Impacted and control slabs were hemisected and one hemisphere was placed into Golgi-Cox solution to incubate in the dark as previously described [31]. The Golgi-Cox solution was replaced with fresh solution after the first 24 hours of the 2-week incubation. After the 2-week incubation, the tissue was washed in a series of distilled H_2_O and increasing sucrose concentrations (10%, 20%, 30%). Slabs were sectioned at 200µm on a vibratome, with a minimum of 5 slices taken from each slab. The development of the Golgi-Cox staining for visualization has been previously described [31].

### Neuron reconstruction and dendrite quantification

Based on the particle tracking and the placement of the triangular elements, a map of regions of interest (ROI) was developed and used to guide the reconstruction of pyramidal neurons within specific areas of the porcine cortex (Fig 1). On average, each brain slab contained five ROI’s, with one neuron being fully reconstructed. Reconstruction was performed in triplicate for each individual brain, resulting in 15 reconstructed pyramidal neurons per brain. A total of 11 slabs (6 control, 5 impact) had adequate Golgi-Cox staining required for accurate neuron reconstruction. Neurons were chosen within each ROI based on the following set of criteria [31]: 1) total stain impregnation in the neuron, 2) staining had to be uniform throughout the neuron, 3) the soma had to be within the 200µm section depth, 4) the neuron had to relatively isolated, or dendritic processes had to be clear enough to follow if there were adjacent cells.

Pyramidal neurons were visualized using a MBF CX9000 camera, mounted on to an Olympus Bx51 microscope at 100x magnification. Neurolucida 360 software (MBF Bioscience, Williston, VT) was used for neuron reconstruction. In brief, the cell body was initially traced, and apical and basal dendrites were traced individually. A minimum of two basal dendrites and one apical dendrite were traced per neuron. Dendritic spines were identified and marked accordingly as tracing took place. Spines were classified either as thin, stubby, or mushroom-like based on their appearance along the dendrite [31,32]. Although this method of spine quantification may underestimate the total number of spines due to the lack of 3D reconstruction and bias of spine type that are parallel to the plane of tissue sectioning, it allows for the direct comparison between the different groups when analyzed [32].

A summary of each traced neuron was collected and analyzed using Neurolucida Explorer (MBF Bioscience, Williston, VT). The apical dendrite spine density was calculated by dividing the total number of spines counted by the total length of the traced dendrites and presented as spines/10µm. Dendritic spine types were normalized by dividing the number of a specific spine phenotype by the total number of spines counted, giving a proportion of dendritic spines. The same analysis was performed on the basal dendrite. Dendritic spine type density was calculated by dividing the total number of specific spine type (mushroom, thin and stubby) by the total length of the traced dendrites. Neuron complexity was analyzed using Neurolucida Explorer (MBF Bioscience, Williston, VT.).

### Statistical analysis

The data are presented as mean ±  standard deviation (SD) for dendritic spine density, spine type density and proportion, and neuron complexity. Total dendritic spine density was compared using a fixed factor analysis of variance (ANOVA; GraphPad Prism 8.0) comparing the mean spine density of neurons in the ROIs for both the apical and basal dendrites. Individual spine type densities and proportions were compared using a 2-way fixed factor ANOVA, with spine type for each individual neuron being normalized to the total spines (mushroom, thin and stubby) counted for the corresponding neuron. This produced a percentage of spine type for each neuron, which was compared across condition and location within the cortex. Neuron complexity was analyzed with a 3-way fixed factor ANOVA (GraphPad Prism 8.0), comparing the mean neuron complexity in the ROIs for both the apical and basal dendrites and between impacted and control conditions. Investigators performing neuronal quantification were blinded. An alpha level of p <  0.05 was considered statistically significant.

## Results

### Dendritic spine density after impact

**Fig 2:**
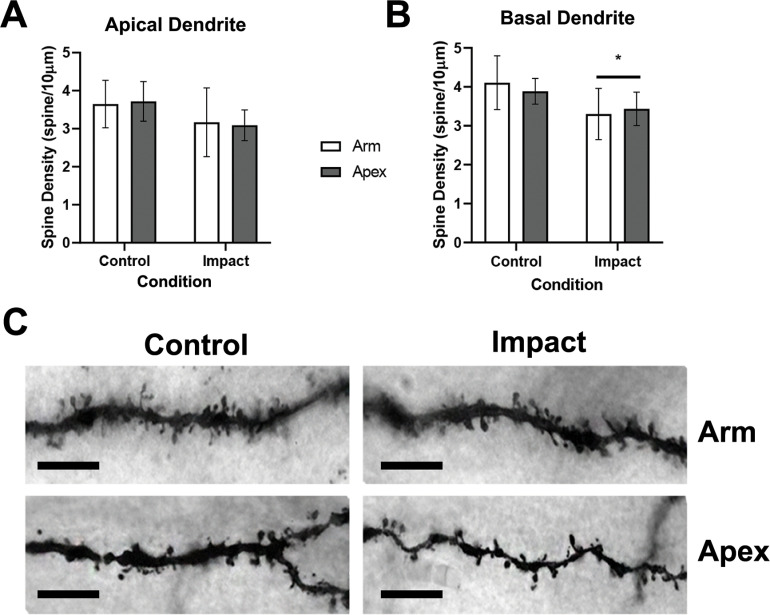
Dendritic spine density in both apical and basal porcine cortical pyramidal cells 1 hour after impact. A) Mean spine density (spine/10µm) along apical dendrites. The apical dendrite spine density did not reach statistical significance (p =  0.17) comparing control and impact conditions. B) Mean spine density along basal dendrites. There was a significant decrease in spine density along the basal dendrites 1 hour after impact ( * =  **p** <  0.05). C) Representative image of Golgi-Cox-stained dendritic spines along the basal dendrites at 100x magnification. Scale bar =  10µm. These data are expressed as mean ±  SD.

To characterize spine type characteristics 1 hour after impact, we investigated several features on both the apical (Fig 2A) and basal (Fig 2B) dendrites of porcine cortical pyramidal neurons. These features included spine density, spine type densities and proportions, and neuron complexity within the depths of the sulci and the adjacent arm of the sulci (Fig 2C). A 2-way fixed factor ANOVA (sulcus location, condition) revealed no significant difference between conditions (control, impact; F [1,16] =  1.998, p =  0.17) and sulcus location (arm, apex) in the overall apical spine density (F [1,16] =  0.081, p =  0.77; Fig 2A). Along the basal dendrite, there was a significant difference for condition (F [1,16] =  4.665, p =  0.046), but no significant difference for sulcus location (F [1,16] =  0.047, p =  0.83; Fig 2B).

### Apical dendrite spines after impact

**Fig 3:**
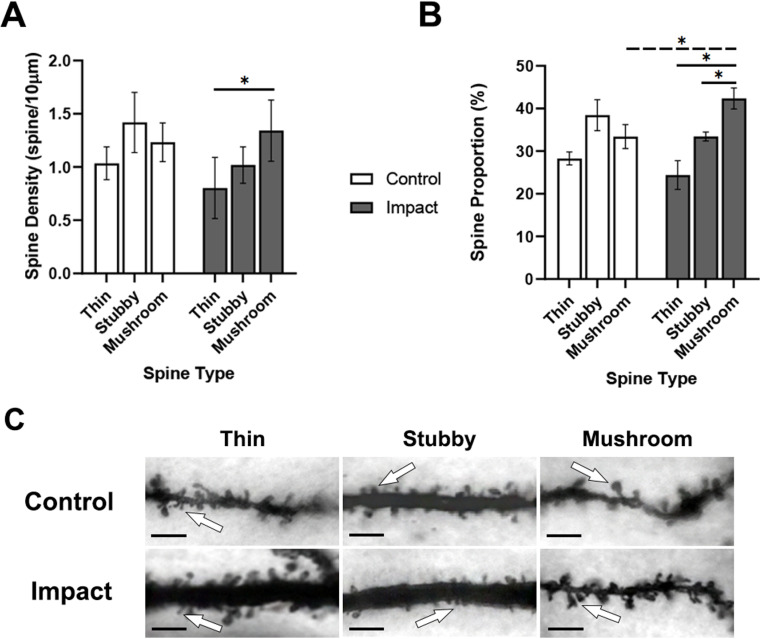
Dendritic spine proportions along the apical dendrite shifting towards mushroom-type spines 1 hour after impact. A) Mean spine type densities. B) Mean spine proportions for apical dendrites showed a shift towards mushroom-type spines (solid line =  within-group differences, dashed line =  between-group differences). C) Representative images of different spine morphology for spine type classification (white arrow indicates example of corresponding spine type). Scale bar in C) =  5µm. *  represents **p** <  0.05. Data are expressed as mean ±  SD.

To assess spine morphology along the apical dendrite of porcine pyramidal neurons, dendritic spine type densities (Fig 3A) and individual spine type proportions (Fig 3B) were quantified 1 hour after impact. A 3-way ANOVA revealed no interaction (F [2,48] =  0.17, p =  0.84), and no effect of sulcus location on either condition (F [1,48] =  0.01, p =  0.91), or spine type densities (F [2,48] =  0.05, p =  0.94). Therefore, sulcus location was not used as a factor. A 2-way ANOVA investigating spine type densities (thin, stubby, mushroom; Fig 3C for representative images) and condition (control, impact) revealed no interaction between spine type densities and condition (F [2,24] =  3.067, p =  0.065; Fig 3A). There was a significant main effect for spine type densities (F [2,24] =  6.95, p =  0.004) but not for condition (F [1,24] =  4.12, p =  0.053). Further analysis of the main effect of spine type density using an ANOVA revealed significant higher density of mushroom-type spine (1.34 spines/10µm) to thin-type spine densities (0.80 spines/10µm) within the impacted group (p =  0.004). There was no observed difference between impacted stubby spines and mushroom, nor within the control group.

A 3-way ANOVA revealed no interaction (F [2,48] =  0.64, p =  0.53), and no effect of sulcus location with either condition (F [1,48] =  0.003, p =  0.95) or spine type proportions (F [2,48] =  0.17, p =  0.84). Therefore, sulcus location was not included as a factor. A 2-way ANOVA investigating apical spine type proportions and condition revealed a significant interaction between spine type proportions and condition (F [2,24] =  21.52, p <  0.0001; Fig 3B). There was a significant main effect of spine type (F [2,24] =  54.81, p <  0.0001) but not condition. A Tukey’s *post hoc* analysis revealed a greater proportion of mushroom-type spines (42.34%) compared to both thin-type (24.39%) and stubby-type spines (33.43%) within the impact condition, as well as compared to control mushroom-type spines (33.41%).

### Basal dendrite spines after impact

**Fig 4:**
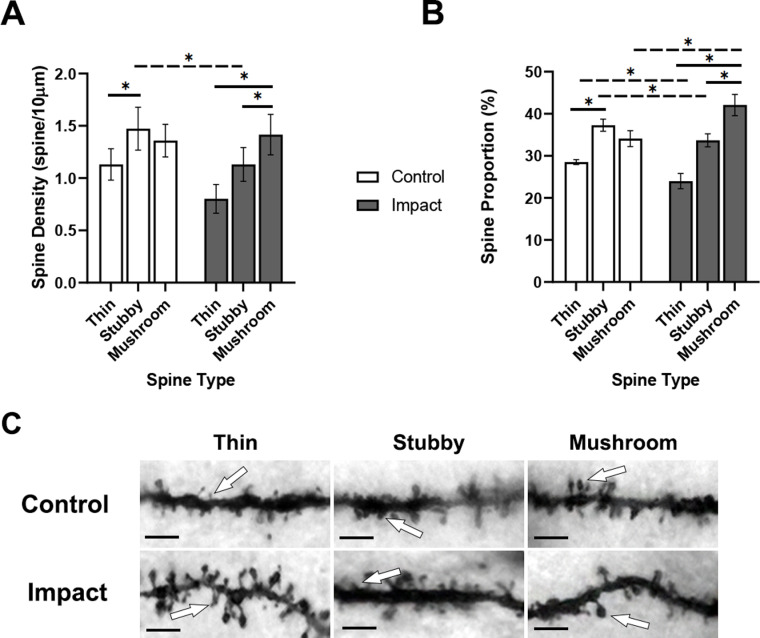
Dendritic spine proportions along the basal dendrite shifting towards mushroom-type spines 1 hour after impact. A) Mean spine type densities. B) Mean spine proportions for basal dendrites shifted towards mushroom-type spines in both the arm and apex of porcine cortical tissue (solid line =  within-group differences, dashed line =  between-group differences). C) Representative images of different spine morphology for spine type classification (white arrow indicates example of corresponding spine type). Scale bar in C) =  5µm. *  represents **p** <  0.05. Data are expressed as mean ±  SD.

To assess spine morphology along the basal dendrite of porcine pyramidal neurons, dendritic spine density (Fig 4A) and individual spine type proportions (Fig 4B) were quantified 1 hour after impact (Fig 4C for representative image). Similar to the apical dendrite analysis, a 3-way ANOVA revealed no interaction (F [2,48] =  0.21, p =  0.81), and no effect of sulcus location on condition (F [1,48] =  1.4, p =  0.24) or spine type (F [2,48] =  0.17, p =  0.85) for basal dendrite spine density. For basal dendrite spine proportion, there was no 3-way interaction (F [2,48] =  1.635, p =  0.21), and no effect of sulcus location on condition (F [1,48] =  0.003, p =  0.95) or spine type (F [2,48] =  0.72, p =  0.49) on proportion; therefore, sulcus location was collapsed and not used as a factor in subsequent analyses. A 2-way ANOVA investigating spine type and condition revealed a significant interaction between individual spine type density and condition (F [2,24] =  4.508, p =  0.022; Fig 4A). There were significant main effects for spine type densities (F [2,24] =  17.3, p <  0.0001) and condition (F [1,24] =  11.03, p =  0.003). A Tukey’s *post hoc* analysis between-groups revealed a higher density of control stubby-type spines (1.47 spines/10µm) compared to impacted stubby-type spines (1.13 spines/10µm). There was a noticeable difference between thin-type spine densities in control (1.132 spines/10µm) and impacted (0.8 spines/10µm) slabs; however, this failed to reach statistical significance (p =  0.0506). Analysis of within-group differences revealed a significantly greater density of mushroom-type spines (1.46 spines/µm) to thin-type spines (0.8 spines/10µm) and stubby-type spines (1.13 spines/10µm) within the impacted group, and a higher density of stubby-type spines (1.47 spines/10µm) to thin-type spines (0.8 spines/10µm) within the control group.

A 2-way ANOVA investigating basal spine type proportions and condition revealed a significant interaction between spine type and condition (F [2,24] =  40.54, p <  0.0001; Fig 4B). There was a significant main effect of spine type (F [2,24] =  128.7, p <  0.0001) but not condition. A Tukey’s *post hoc* analysis between-groups found a significantly higher proportion of impacted mushroom-type spines (42.1%) compared to control (34.09%), a significantly lower proportion of impacted thin-type spines (24.0%) compared to control (28.51%), and a significantly lower proportion of impacted stubby-type spines (33.68%) compared to control (37.29%). When analyzing within-group differences, there was a significantly higher proportion of mushroom-type spines (42.1%) compared to both thin-type (24.0%) and stubby-type spines (33.68%) within the impact condition.

### Pyramidal neuron complexity after impact

**Fig 5:**
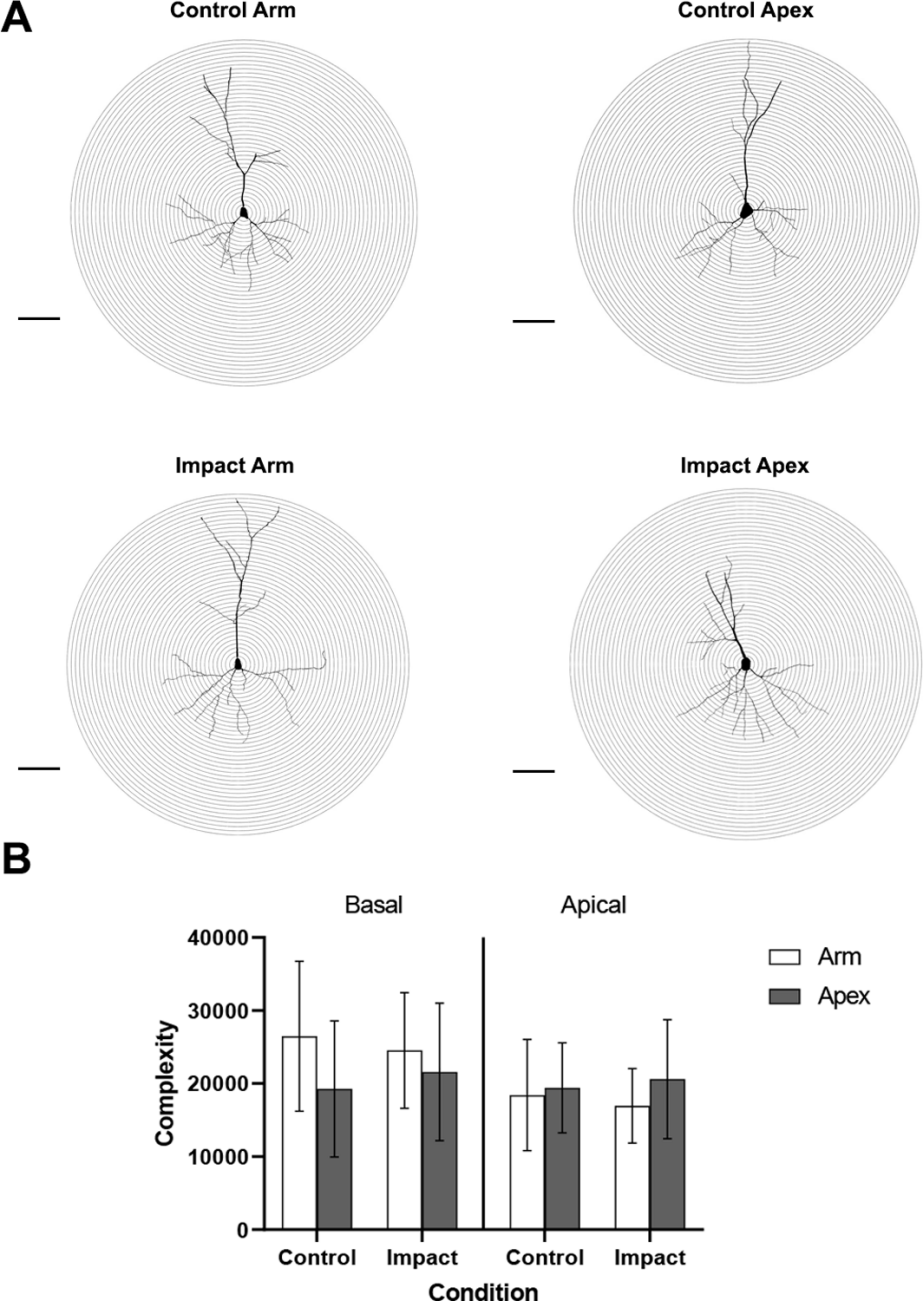
Cortical complexity measurements of both basal and apical dendrites within the porcine cortex. A) Schematic representation of pyramidal neurons in the arm and apex of both conditions. Scale bar =  100µm, while each concentric ring is increasing radii by 10µm from the soma. B) Mean complexity measurements were similar across groups and conditions 1 hour after impact. These data are expressed as mean ±  SD.

Cortical neuron complexity is based on the dendritic complexity index, evaluating parameters such as dendritic length, number of branch points and tips, and number of primary dendrites (see Fig 5A for representative images of pyramidal neurons from each group [33]). A 3-way ANOVA comparing dendrite, location, and condition (Fig 5B) revealed no change in neuron complexity between conditions, type of dendrite or ROIs 1 hour after impact.

### Dendritic spine proportions vs. strain after impact

As described in the methodology section Particle Tracking and Strain Analysis, displacement of the markers positioned within the tissue specimens were tracked in the HSXR images and used to calculate the local strain response where the dendrites were analyzed. The 2D Green-Lagrangian strains were calculated from a set of 3-node triangular elements within different areas of the porcine cortex, as seen for each tissue sample in Fig 1. On average, 5 nodes (regions of interest; ROI) were chosen per brain slab and the strain response was defined by the maximum and the minimum principal strains.

**Fig 6:**
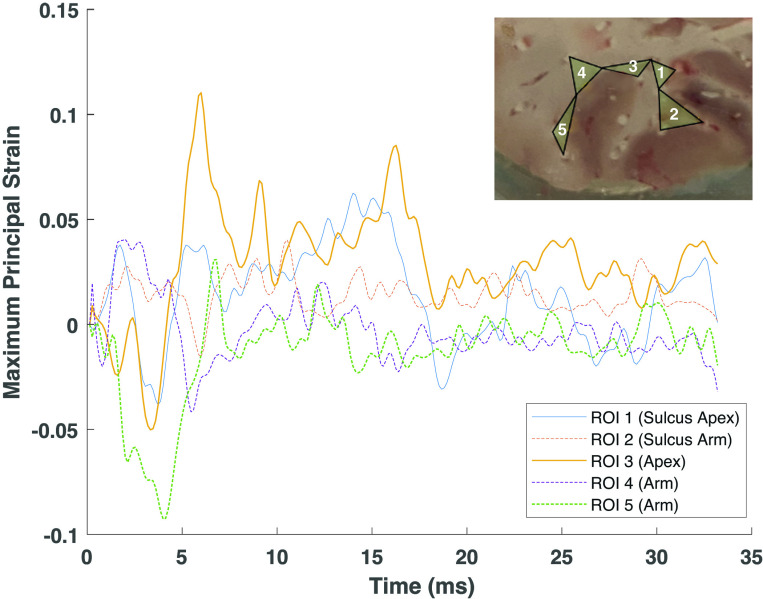
An example of maximum principal strain (MPS) response for *ex vivo* porcine brain tissue sample 5 with inset image showing the locations of the 3-node triangular elements (ROI) used in the calculations.

**Fig 7:**
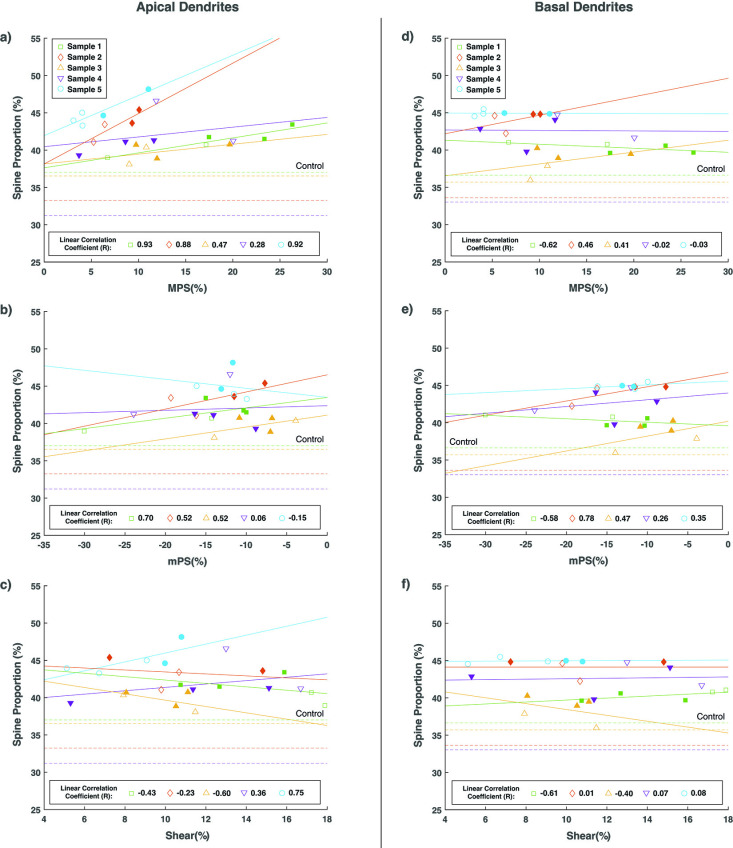
Proportion of mushroom-type spines in the apical dendrites plotted against A) MPS, B) mPs, and C) maximum shear strain. Proportion of mushroom-type spines in the basal dendrites plotted against D) MPS, E) mPS, and F) maximum shear strain. Each marker represents a different region measured within the cortex of the 5 slabs evaluated. Solid markers represent regions corresponding to the arm location of the sulcus, whereas unfilled markers represent the apex location regions. The percentage spine proportion of the control porcine coronal slabs have been plotted as dashed lines in the same color as the corresponding impact slabs.

A plot of the MPS response for each ROI in slab sample 5 is shown as an example in Fig 6. The peak strain values were plotted against the spine type proportion and the individual spine type densities in Fig 7. In these plots, each point represents the data from an individual ROI, while ROIs from the same brain slab are grouped together as a single sample (Fig 6). The plots included in-specimen comparisons among various cortical locations from the sulcus arm and apex locations, as baseline spine type distributions may differ between individual brains. While all impact loads resulted in an increase in the mushroom-type spine proportions, these changes were proportional to the level of the tensile load within the ROI.

The apical and basal dendrite mushroom-type spine proportions in each ROI was plotted against the peak tensile (MPS), peak compression (mPS) and maximum shear strain (Fig 7). While the mushroom-type morphologies increased in proportion with increasing MPS in all samples, the density did not increase in tandem. Thus, it appears that the increase in mushroom-type spine proportions did not arise from the increased prevalence of mushroom-type morphologies, but rather a shift away from other spine type morphologies (e.g., thin, and stubby) with increasing tension.

### Average strain in sulcus location after impact

**Fig 8:**
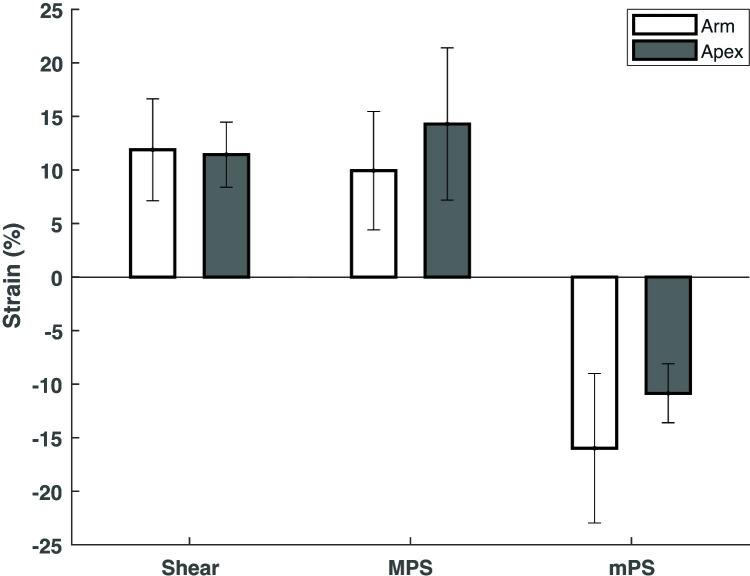
The total average tensile, compressive and shear strain in the regions corresponding to the arm and the apex locations of the sulcus. These data are expressed as mean ±  SD.

To assess the influence of sulcus location on the observed average strain post-impact, the average tensile, compressive and shear strain in the regions corresponding to the sulcus arm and apex locations were compared (Fig 8). The average tensile response in the apex was higher compared to the arm locations, although the difference failed to reach statistical significance (Fig 8). The higher tensile strain response at the apex of the sulcus is consistent with prior experimental [25,27] and computational results [34,35] relating to the influence of the gyrencephalic and the anisotropic structure of brain tissue.

There were no clear trends between the sulcus arm and apex locations and the spine morphology. This may imply that the morphological changes of the dendritic spine are not sensitive to the sulcus location, but further investigation is required to confirm this (Fig 7).

## Discussion

Dendritic spines are in a constant state of change, with spine phenotypes responding to both the surrounding extracellular environment and the intraneuronal environment [36,37]. In the present study, we observed a shift in the distribution of thin- stubby- and mushroom-type spines following impact compared to the distribution of spine types in controls. This change after impact seemed to contribute to an increased proportion of mushroom-type spines that was shown to be sensitive to increasing tensile loads in the apical dendrites. Similar results of changes in thin- and stubby-type spines resulting in an increased proportion of mushroom-type spines have been observed in different models of neuropathology [32,38]. Numerous reports have shown that the size of the spine head and spine morphology are well correlated to the strength and stability of the synapse [39–43]. This showed that higher levels of tensile strain may cause changes in synaptic response, which is consistent with the prevailing theories linking post-concussive symptoms to higher MPS within the brain [44–48]. Dendritic spine injury probability has been shown to be linked to MPS values calculated in finite element modeling [48] meaning that dendritic spines exposed to higher MPS are at a higher risk of injury and damage. As thin-type spines have been shown to be unstable compared to the mushroom-type spines [32], they may be destroyed further with increasing MPS, while the mushroom-type spines are able to withstand the increased tensile load due to their stable nature.

The custom drop tower apparatus allowed for consistent linear impact loading of the brain tissue. While this mode of loading does not include a rotational acceleration component, which is commonly correlated to diffuse brain injury, we are using this approach to directly compare measured strain to the related changes in the synapses. Since the brain is now unrestricted by the confines of the skull, the conditions at its boundaries are altered, and the way tissue deformation occurs no longer has the same direct connection to the head’s acceleration pattern as in a closed-head impact scenario. Therefore, it is important to note that the purpose of this investigation was not to reproduce a certain type of TBI loading, but rather to subject the brain tissue to a quantifiable impulsive strain load. Additionally, this model does not capture *in vivo* physiological conditions, such as, but not limited to, blood flood and intercranial pressure. This model does, however, provide insight into how the unique material properties of the gyrified brain, which are difficult to replicate *in vitro* [49], respond to impact forces and how neurons respond to these forces.

Given that thin-type and stubby-type spines are unstable compared to mushroom-type spines [43], the shift in a higher proportion of spine types to mushroom after impact could be due to the impact disrupting thin- and stubby-type connections, with the larger mushroom-type spines having stronger synaptic connectivity properties. The properties that maintain mushroom-type connections could be the presence of adhesion molecules, the size of the post-synaptic density and large clusters of f-actin filaments making the synapse less sensitive to environmental change [40,43]. Further research into the molecular mechanisms involved will provide insight into how mushroom-type spines maintain connectivity within the hyperacute phase after brain impact.

This increase in proportion of the mushroom-type phenotype is interesting, as this phenotype is associated with excitatory signaling and synaptic activity between neighbouring neurons [50,51]. Large, mushroom-type spines have been reported to have high glutamate sensitivity and may further promote this phenotype through maladaptive use of the same synaptic mechanisms involved in long-term potentiation (LTP) during neuropathological conditions [52]. During the hyperacute phase after impact (minutes to hours), the adjacent extracellular environment around the neuron becomes excitatory, as evident from an increase in extracellular glutamate and Ca^2+^ that remain elevated for hours [53,54]. Glutamate and Ca^2+^ play a role in mediating changes in dendritic spine morphology into mushroom-type spines through the activation of NMDA receptors along the post-synaptic terminal [55]. Moreover, glutamate and Ca^2+^ have neurodegenerative characteristics if not properly cleared or buffered [53]. Prolonged glutamate presence within the synaptic cleft can continuously activate the NR2B subunit of NMDA receptors, causing increased Ca^2+^ to enter the post-synaptic neuron and activation of neurodegenerative pathways [56]. Using the controlled cortical impact model in rodents, Pijet et al. [21] observed increased matrix metalloproteinase-9 (MMP-9) levels within the first 30 minutes after impact and remained elevated for days following impact. In a follow up study Pijet et al. [23] observed that after 24 hours, the elevated expression of MMP-9 was associated with dendritic spines that were shorter and wider. The prolonged synaptic mechanisms involved in LTP and favouring of mushroom-type spines could be due to the imbalance of extracellular excitotoxic conditions and the increased excitatory conditions within the synapse and the neuron.

Cortical complexity is calculated using parameters such as length, branch points and endings, and number of primary dendrites [33]. This index provides an understanding of how the dendrites react to the environment around them. Since cellular changes during the hyperacute phase are localized to individual neurons, and the development of regional cortical impairments and dendritic retractions can take weeks to months to develop [2], our finding of no change to cortical complexity supports this statement. Dendrite degeneration has been observed as early as several days after controlled cortical impact (CCI) and fluid percussion injury (FPI) in rodents, with reduction in spines and function mediating degenerative conditions [57,58]. The degeneration of dendrites and dendritic spines has been shown to persist for months after impact, reducing the amount of mushroom-type spines and impairing cognitive function [59]. The promotion of mushroom-type spines, while the neurons show no reduction in complexity in the present study could help explain the hyperexcited state that is observed shortly after impact [60], promoting the activation of neurodegenerative pathways [59,61].

## Conclusion

To summarize, in the present study, porcine brain tissue underwent a linear drop impact from a height of 0.9m. After impact, the tissue was left to incubate for 1 hour to let hyperacute cellular responses take place. Using Golgi-Cox reconstruction, it was revealed that within 1 hour after impact there was a preference for excitatory conditions, as evident through the increased proportion of mushroom-type spines. The change in spine morphology was proportional to tensile load applied to porcine brain tissue along the apical dendrites. These findings give insight into the link between impact-induced tensile strain and the promotion of excitatory conditions within the hyperacute phase post-impact. The promotion of excitatory conditions within the hyperacute phase also provides insight into the activation of excitotoxic cascades that occur later after brain impact.
